# Ex vivo preliminary investigation of radiographic quantitative assessment of cranial tibial displacement at varying degrees of canine stifle flexion with or without an intact cranial cruciate ligament

**DOI:** 10.1186/s12917-018-1599-5

**Published:** 2018-09-03

**Authors:** Katrina A. Castaneda, Caleb C. Hudson, Brian S. Beale

**Affiliations:** 1 MedVet Medical and Cancer Centers, 300 E. Wilson Bridge Rd, Worthington, OH 43085 USA; 2 Gulf Coast Veterinary Specialists, 1030 Wirt Rd, Houston, TX 77055 USA

**Keywords:** Canine stifle, Cranial cruciate ligament injury, Cranial Tibial subluxation, Radiography

## Abstract

**Background:**

The presence of cranial tibial subluxation can aid in the detection of joint instability as a result of CrCL injury. Detection of cranial tibial subluxation has been described using the tibial compression test (TCT) and cranial drawer test (CDT); however, diagnosis of CrCL insufficiency by assessing cranial subluxation motion of the tibia is subjective and difficult to quantify accurately. The aim of this study was to investigate a measurement technique to assess the degree of cranial tibial displacement relative to the femoral condyles on mediolateral projection stifle radiographs at varying degrees of stifle flexion (90°, 110°, and 135°) in CrCL intact, partially, and completely transected conditions. Radiographic measurements included: CrCL length and intercondylar distance (ICD), defined as the distance between the tibial mechanical axis (TMA) and the femoral condylar axis (FCA). The influence of CrCL status, stifle flexion angle, and measurement type on measured distance was evaluated. The relationship between CrCL length and ICD measurement was also assessed.

**Results:**

Complete transection of the CrCL resulted in significant cranial tibial displacement. Stifle flexion angle affected ICD, but not CrCL length. Normalized measured CrCL length and ICD were significantly different; however, no differences existed between the change in distance detected by CrCL length and ICD measurements as CrCL transection status changed. Correlation coefficients detected a significant positive correlation between measured CrCL and ICD.

**Conclusion:**

The ICD measurement technique was able to quantify tibial displacement at various stifle flexion angles in the intact and completely transected CrCL conditions. The ICD measurement was more affected by stifle flexion angle than was the CrCL length.

**Electronic supplementary material:**

The online version of this article (10.1186/s12917-018-1599-5) contains supplementary material, which is available to authorized users.

## Background

Cranial cruciate ligament (CrCL) rupture is a common orthopedic disease in dogs [1, 2]. CrCL insufficiency leads to stifle instability, which results in cranial subluxation of the tibia relative to the femur during joint loading [3]. Stifle joint instability results in lameness and the development of osteoarthritis [4, 5]. The CrCL consists of 2 functional components: a craniomedial band and a caudolateral band [6, 7]. The character and severity of clinical signs resulting from CrCL injury varies to some extent based on whether one or both bands of the CrCL are torn (partial versus complete CrCL rupture) [7, 8]. Transection of the craniomedial or caudolateral band of the CrCL has been shown to cause mild joint instability with ≤3 mm of cranial tibial translation with flexion of the stifle [7].

Physical and radiographic examination techniques are utilized most commonly to diagnose CrCL deficiency. The presence of cranial tibial subluxation can aid in the detection of joint instability as a result of CrCL injury [9]. Detection of cranial tibial subluxation has been described using the tibial compression test (TCT) [10] and cranial drawer test (CDT) [11]. However, diagnosis of CrCL insufficiency by assessing cranial subluxation motion of the tibia is subjective and difficult to quantify accurately. Previous studies have questioned the reliability of the TCT [11, 12]. In a study by Carobbi and Ness (2009), the TCT had a sensitivity of 64% and a specificity ranging from 82.4–100% [11]. The TCT and CDT provide a subjective evaluation of cranial tibial translation secondary to CrCL insufficiency, but these tests do not objectively quantify the magnitude of cranial tibial translation that occurs during manipulation of the stifle. Several techniques for quantifying tibial subluxation radiographically have been previously reported, including displacement of the femoral condyles or long digital extensor fossa along a plane parallel to the tibial plateau, implantation of radio-opaque markers at the femoral and tibial attachments of the CrCL, and identification of bone landmarks correlating to the femoral and tibial attachments of the CrCL [9, 13, 14]. These techniques have been demonstrated to accurately quantify cranial tibial translation when utilized properly, but they utilize landmarks that may be difficult to identify in the presence of concurrent osteoarthritis or may not be practical to implement in the majority of clinical patients (radio-opaque marker implantation).

It has been our radiological observation that the center of the femoral condyles is noticeably shifted caudal to the tibial intercondylar eminences on the mediolateral projection radiograph of the stifle in the presence of cranial tibial subluxation when the stifle is positioned at 90°. The purpose of this study was to investigate a measurement technique for assessing the degree of cranial tibial displacement relative to the femoral condyles on mediolateral projection stifle radiographs at varying degrees of stifle flexion (90°, 110°, and 135°) in CrCL intact, partially, and completely transected conditions and comparing results of the investigational technique to the results obtained by measuring the length of the CrCL. We hypothesized that our radiographic measurement technique would allow accurate detection and quantification of the magnitude of tibial displacement relative to the femur at all measured stifle flexion angles and in all CrCL conditions when compared to measured CrCL length.

## Methods

### Joint preparation

**Fig. 1.**
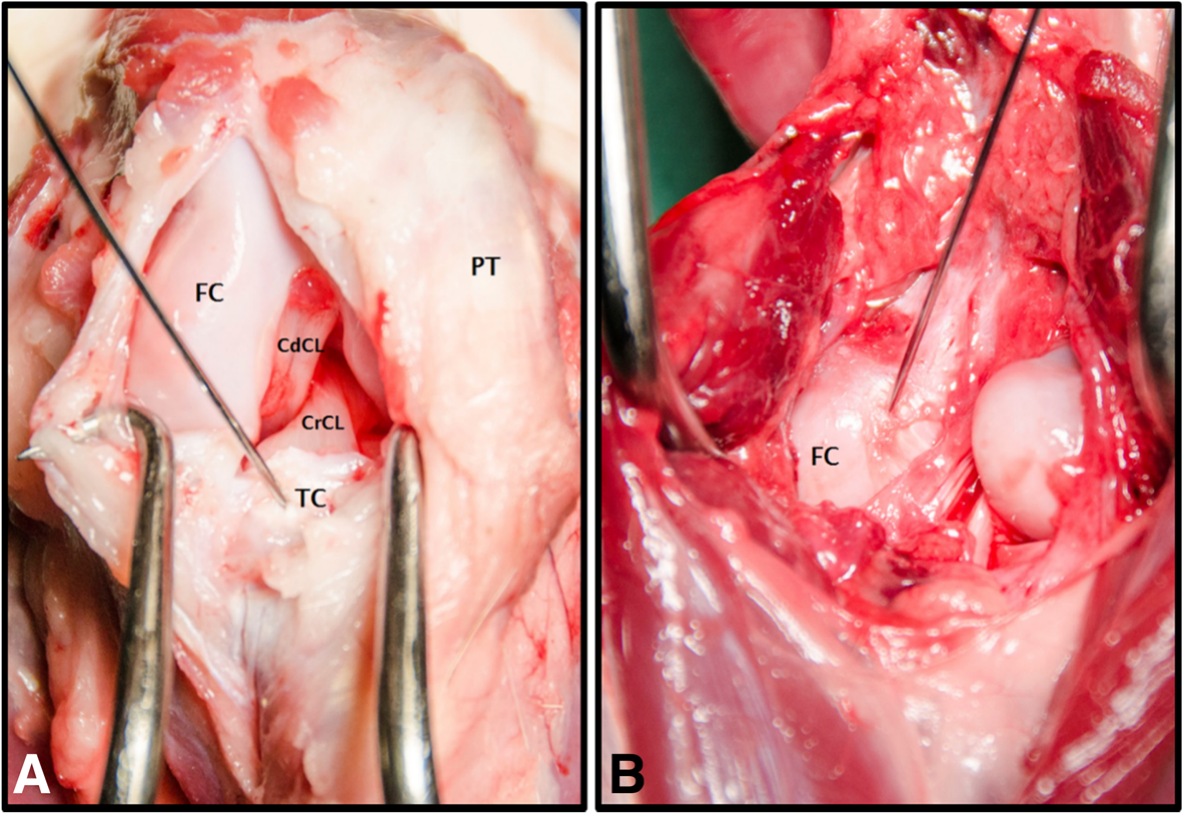
**a** Photograph of a craniomedial approach to the stifle. The Kirschner wire is inserted at the tibial attachment of the CrCL. CdCL: Caudal cruciate ligament. FC: Femoral condyle. PT: Patellar tendon. TC: Tibial condyle. **b** Photograph of a caudal approach to the stifle. The Kirschner wire is inserted at the femoral attachment of the CrCL. FC: Femoral condyle

Eight normal cadaveric pelvic limbs from five, medium to large breed adult dogs euthanized at a local animal shelter for reasons unrelated to this study and without radiographic evidence of stifle disease were included. A body weight of 15–25 kg was required for inclusion in the study. Following euthanasia, the cadavers were stored at 2 °C. Immediately prior to testing, the cadavers were warmed to room temperature (approximately 25 °C) and the hind limbs were disarticulated at the coxofemoral joint. The femoral attachment of the CrCL was exposed via a caudomedial arthrotomy approach. The tibial attachment of the CrCL was exposed via a craniomedial arthrotomy approach. The presence of an intact CrCL with no gross evidence of pathologic changes in the stifle joints was confirmed. Radiopaque fiducial markers were inserted at the femoral and tibial attachments of the CrCL using 0.035 mm Kirschner wires (Fig. 1a, b). Wires were then cut short and countersunk to be flush with the ligament attachment sites. The distance between these markers represented the length of the CrCL. The joint capsule and soft tissue structures were closed in routine fashion using size 3–0 polydioxanone^1^ suture. Throughout specimen preparation and data collection, specimen tissues were kept moist using a saline spray.

### Radiography

**Fig. 2:**
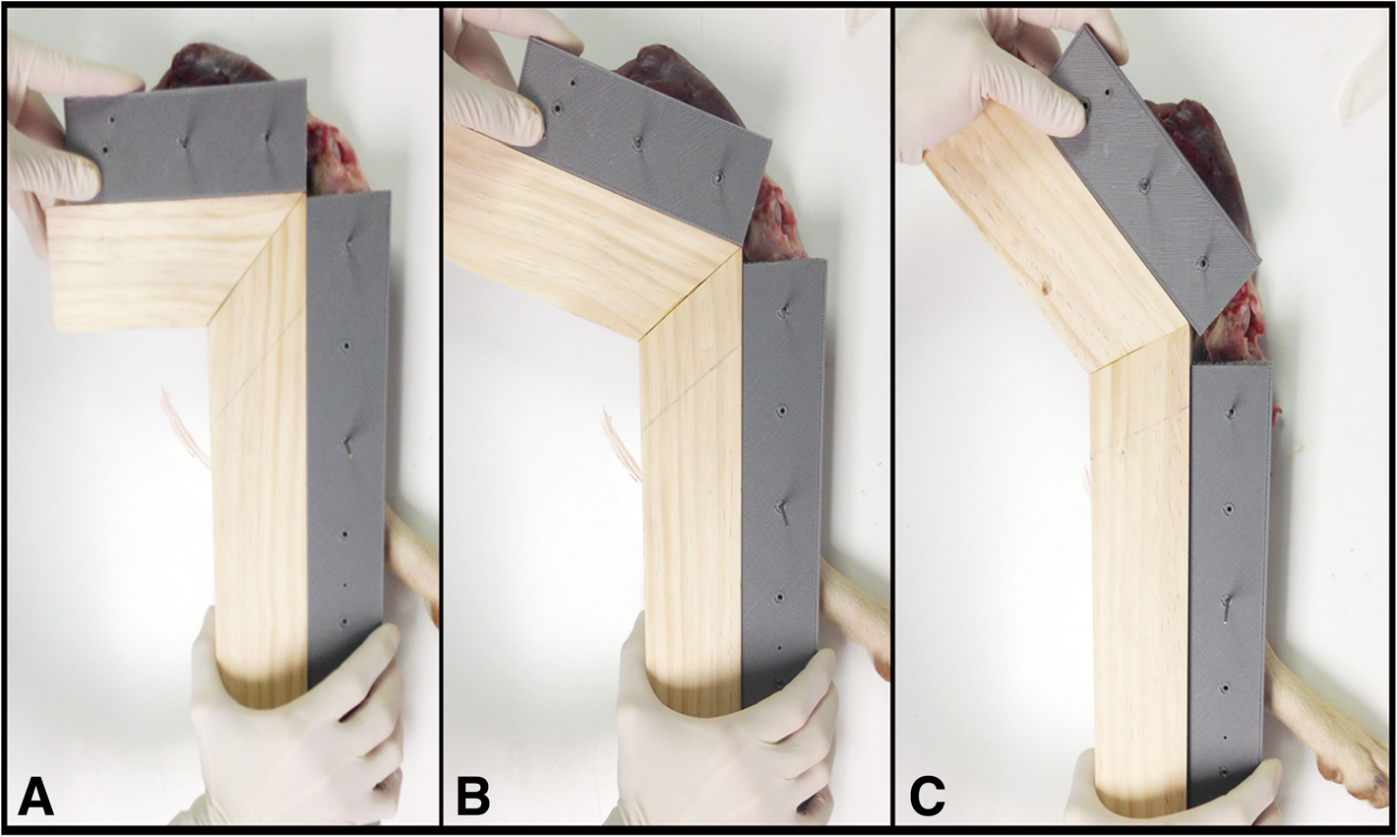
Photograph of a cadaver stifle positioned at 90° (**a**), 110° (**b**), and 135° (**c**) of flexion

Custom fabricated bone alignment guides composed of polylactic acid (PLA) filament^2^ were used to precisely position the stifle at 90°, 110°, and 135° of flexion. Four (two each in the femur and tibia), 2 mm Steinmann pins were inserted in the femoral and tibial diaphyses in the coronal plane from medial to lateral, perpendicular to the long axis of the bone. The pins were then inserted into corresponding holes in the alignment guides. A wooden positioning device was matched to the alignment guides to produce repeatable stifle flexion angles of 90°, 110°, and 135° (Fig. 2). The limbs were positioned with the lateral surface contacting the radiographic table. All radiographs were obtained using a digital radiography system.^3^ The radiographic beam was centered over the stifle and collimated to include the entire tibia and distal half of the femur. Mediolateral projection radiographs of the stifles with intact CrCL were obtained at 90°, 110°, and 135° of flexion while actively flexing the tarsal joint. The manual flexion force applied to the tarsus by the examiner (KC) during radiograph acquisition was designed to mimic the tibial compression test and result in generation of tibial thrust force. The tarsus was flexed until parallel to the femur for radiographs at all 3 flexion angles. All radiographs included a spherical external calibration marker positioned at the level of the stifle joint.^4^ A mini craniomedial arthrotomy was performed. The craniomedial band of the CrCL was then transected using a #11 scalpel blade. Radiographic projections were repeated at all stifle flexion angles after transection of the craniomedial band. The caudolateral band of the CrCL was subsequently transected through the craniomedial arthrotomy, simulating a complete CrCL tear. Radiographic projections were repeated at all stifle flexion angles after transection of the caudolateral band. The craniomedial arthrotomy was closed in routine fashion using size 3–0 polydioxanone^1^ suture following craniomedial band and caudolateral band transections. All radiographs were performed by the same examiner (KC).

### Radiographic analysis

**Fig. 3:**
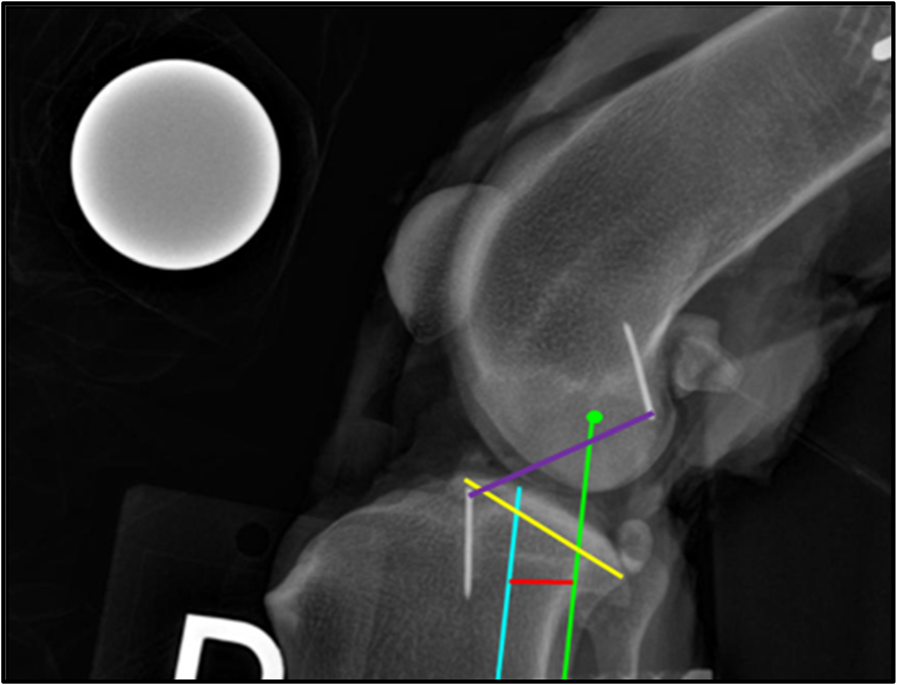
Mediolateral projection radiograph of stifle at 110° of flexion with ICD measurement identified. Purple line: CrCL length (mm), Green dot: center of a best fit circle around the femoral condyles, Blue line: tibial mechanical axis, Green line: femoral condylar axis, Red line: ICD length (mm), Yellow line: tibial medial condyle length (mm)

The radiographic images for each stifle were evaluated by 2 investigators (KC, CH) (non-blinded, non-randomized). Radiographic measurements were completed using digital radiographic templating software.^5^ The tibial mechanical axis (TMA) in the sagittal plane was identified as a line intersecting the center of a best fit circle applied to the talus and the midpoint of the intercondylar eminences, as described by Dismukes and colleagues [15]. If the intercondylar eminences were not exactly superimposed, the midpoint of each eminence was identified. The TMA was drawn through a point midway between the two individual eminence midpoints. A best fit circle was applied to the femoral condyles. A femoral condylar axis line (FCA) was defined as a straight line parallel to the TMA intersecting the center of the condylar best fit circle. If the femoral condyles were not exactly superimposed, a best fit circle was drawn around each femoral condyle and the center of each condyle was identified. The FCA intersection was then defined as the midpoint between the centers of the two best fit circles. To quantify the magnitude of cranial tibial displacement, the distance between the TMA and the FCA was measured and recorded as the intercondylar distance (ICD). The distance between the fiduciary markers at the CrCL attachments was measured and recorded as the length of the CrCL. The length of the medial tibial condyle was also measured (Fig. 3).

### Statistical analysis

All measurement data were recorded into electronic spreadsheets^6^ and statistical tests were carried out using commercial software.^7^ The measured CrCL length and ICD measurement were normalized using the length of the medial tibial condyle to account for differences in patient size. Univariate and multivariate ANOVA (Analysis of variance) tests were used to evaluate the influence of CrCL status, stifle flexion angle, and measurement type on measured distance. Normalized CrCL length and ICD measurement values were averaged at each stifle angle to analyze the change in distance from intact to partial and partial to complete CrCL status (see Additional file 1: Table S1), then relationships were evaluated using univariate and multivariate ANOVA tests. The relationship between CrCL length and ICD measurement was assessed by calculation of Pearson’s correlation coefficient for each data set (intact, partially transected, and completely transected status of the CrCL) at each stifle flexion angle (90°, 110°, and 135°). To assess the relationship between normalized ICD measurement and normalized CrCL length, the data from both observers was averaged at all examined stifle flexion angles. Inter-observer agreement of normalized CrCL length and ICD measurements was assessed by calculation of Pearson’s correlation for the two investigators where 0 represented no agreement and 1 represented perfect agreement. Inter-observer reliability was determined for both investigators (KC, CH) using the Pearson’s correlation coefficient for normalized CrCL length and ICD measurements. An inter-observer Pearson’s correlation coefficient value of r greater than 0.74 was considered excellent, *r* = 0.60–0.74 good, *r* = 0.40–0.59 fair, and r less than 0.40 was considered poor. *p* < 0.05 was set as significant for all analyses. Results are reported as mean ± standard deviation (SD).

## Results

### Specimens

Eight stifles from 5 dogs were included. Three female dogs and 2 male dogs with a mean ± SD body weight of 20 ± 3.4 kg were used. The breeds represented were Labrador Retriever (*n* = 2), German Shepherd (*n* = 2), and Mixed Breed Dog (*n* = 1). Five left pelvic limbs and 3 right pelvic limbs were used in this study.

### Radiographic measurement of cranial tibial subluxation

#### Normalized CrCL length

**Fig. 4:**
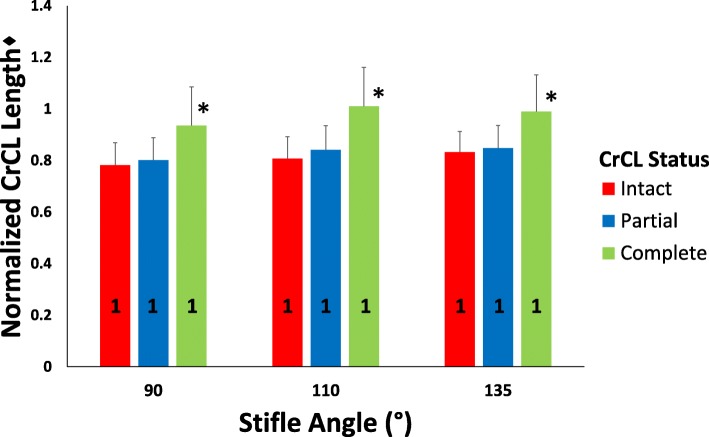
Bar graph of mean ± SD normalized CrCL length values as CrCL status sequentially changes at each stifle angle. Univariate ANOVA test performed to assess for differences in normalized mean CrCL length as CrCL status changes. Presence of an asterisk (*) indicates a significant difference (*P* < 0.05) between CrCL length values within a single stifle angle group. Presence of a different numeral (1, 2, or 3) on a bar indicates a difference between stifle angle groups within a single CrCL status. ♦The CrCL length measurements were normalized using the length of the medial tibial condyle

**Fig. 5:**
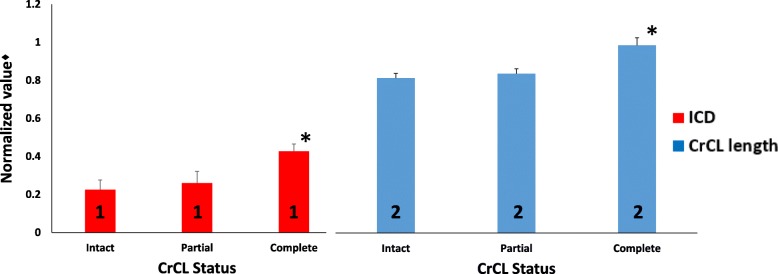
Bar graph of group mean ± SD normalized CrCL length and ICD values as CrCL status changes from intact to complete. All stifle flexion angles are pooled. Univariate ANOVA tests performed to assess for differences in group means of both normalized CrCL length and ICD means as CrCL status changes, as well as to assess for differences between ICD measurement and CrCL length measurement within a single CrCL status. Presence of an asterisk (*) indicates a statistically significant difference (*P* < 0.05) between group means within a measurement type. Presence of a different numeral (1, 2, or 3) on a bar indicates a difference between ICD measurement and CrCL length measurement within a single CrCL status. ♦The CrCL length and ICD measurements were normalized using the length of the medial tibial condyle

Complete transection of the CrCL significantly increased normalized CrCL length (*P* < 0.001), while partial transection of the CrCL did not increase measured normalized CrCL length (*p* = 0.326) at all stifle angles as shown in Fig. 4. Stifle angle did not significantly affect normalized CrCL length (*P* = 0.150) (Fig. 4). Combined stifle angle measurements demonstrated a mean ± SD increase in the measured length of normalized CrCL from intact to partial CrCL transection of 0.030 ± 0.044, and from partial to complete CrCL transection of 0.140 ± 0.104 (Fig. 5).

#### Normalized ICD measurement

**Fig. 6:**
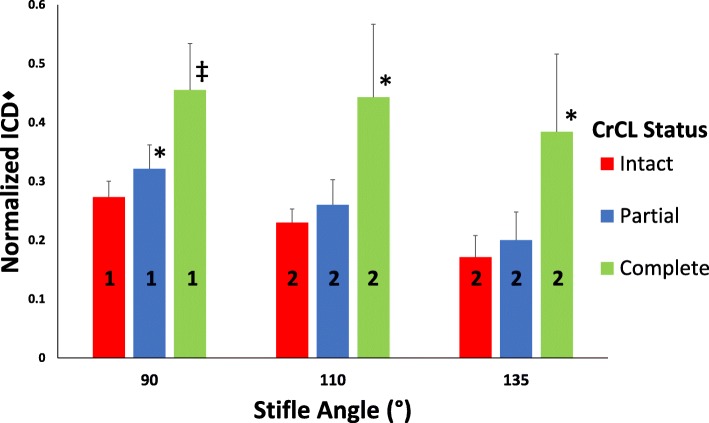
Bar graph of mean ± SD normalized ICD values as CrCL status sequentially changes at each stifle angle. Univariate ANOVA tests performed to assess for differences in normalized mean ICD as CrCL status changes. Presence of an asterisk (*) or double dagger (‡) indicates a significant difference (*P* < 0.05) between normalized ICD measurements within a single stifle angle group. Presence of a different numeral (1, 2, or 3) on a bar indicates a difference between stifle angle groups within a single CrCL status. ♦The ICD measurements were normalized using the length of the medial tibial condyle

Normalized ICD measurement was significantly affected by stifle angle *(P* < 0.001) and CrCL status *(P* < 0.001) (Fig. 6). Complete transection of the CrCL significantly increased normalized ICD *(P* < 0.001*)* at all stifle angles, while partial transection of the CrCL significantly increased normalized ICD at 90° (*P* = 0.019), but not at 110° (*P* = 0.110) or 135° (*P* = 0.183). All measurements taken at the 90° stifle angle are significantly greater than measurements taken at 110° and 135° (*P* < 0.001); however, measurements taken at 110° and 135° were not significantly different (*P* = 0.224) (Fig. 6). Combined stifle angle measurements demonstrated a mean ± SD increase in measured length of normalized ICD of 0.035 ± 0.054 from intact to partial CrCL transection conditions, and 0.166 ± 0.114 from partial to complete CrCL transection conditions (Fig. 5).

#### Correlation between measurements

**Fig. 7:**
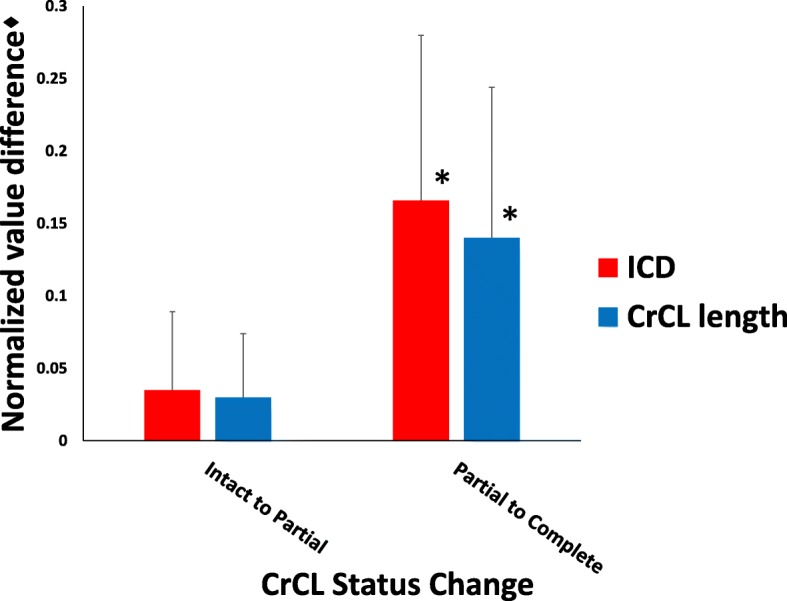
Bar graph of the mean change in measured length ± SD of normalized CrCL and ICD values as CrCL status changes from intact to partial and from partial to complete transection. All stifle flexion angles are pooled. Presence of an asterisk (*) indicates a significant difference (*P* < 0.05) compared to groups without an asterisk. ♦The CrCL length and ICD measurements were normalized using the length of the medial tibial condyle

Normalized measured CrCL length and ICD were significantly different (Fig. 5); however, no differences existed between the change in distance detected by normalized CrCL measurement and normalized ICD as CrCL transection status changed from intact to partial and from partial to complete. The magnitude of the change from partial to complete CrCL status as assessed by both ICD and CrCL length is significantly greater than the magnitude of the change from intact to partial, as reported in the two previous paragraphs (Fig. 7). Calculated Pearson’s correlation coefficients detected a statistically significant positive correlation between CrCL measurement and ICD at all stifle flexion angles (*r* = 0.772 at 90°, *r* = 0.817 at 110°, and *r* = 0.741 at 135°).

#### Inter-observer agreement

The inter-observer Pearson’s correlation coefficients were in excellent agreement with *r* = 0.883 for the normalized ICD measurement and *r* = 0.926 for the normalized CrCL length measurement.

## Discussion

In this study, we evaluated a measurement technique to assess the magnitude of cranial tibial displacement using mediolateral projection stifle radiographs at varying degrees of stifle flexion. Our ICD measurement was determined by measuring the distance between the tibial mechanical axis and an axis we defined as the femoral condylar axis. Our study found the ICD measurement accurately quantified the magnitude of cranial tibial subluxation radiographically at stifle flexion angles of 90°, 110°, and 135°.

Similar to previous reports, we detected a measureable increase in CrCL length with complete transection of the CrCL [14]. Our ICD measurement also increased with complete transection of the CrCL, however, the magnitude of CrCL length measurement was significantly greater than the ICD measurement. This difference between the ICD measurement and CrCL length was expected because the two measurement methods were based on different anatomic points. The ICD measurement is based off of a femoral point that is located cranial to the femoral attachment of the CrCL and a tibial point that is located caudal to the tibial attachment of the CrCL. Neither of the ICD measurement points is located in an isometric position, based on previous studies, while the distance between CrCL attachment points has been shown to remain fairly constant during stifle range of motion [16, 17]. Given these considerations, it was not surprising that we found that the absolute magnitude of measured ICD was different than the CrCL length at all flexion angles and that the ICD measurement was more affected by stifle flexion angle than was the CrCL length. The ICD changes as stifle flexion angle increases because the center of the femoral condyles moves caudally, while the femoral attachment point of the CrCL is a more isometric point and maintains a more constant position [16, 17].

Despite the difference in measured length between the ICD distance and the CrCL length, the magnitude of the change in measured length (which represents the magnitude of cranial tibial displacement) from intact to partial CrCL status and from partial to complete CrCL status was not statistically significant between our ICD measurement and CrCL length. Correlation coefficient values also supported a positive relationship between ICD and CrCL length in the study specimens. These results suggest that ICD is able to accurately quantify the magnitude of cranial tibial displacement, but is not an appropriate measurement of CrCL length.

Partial CrCL ruptures often cause hindlimb lameness and stifle pain and have been reported to be present with a prevalence of approximately 8% of the population of surgically treated dogs with cranial cruciate ligament disease based on the results of one study performed by Scavelli and colleagues [8, 11]. The craniomedial band of the CrCL is taut in both flexion and extension, while the caudolateral band is taut in extension, but lax in flexion [13]. In our study, the craniomedial CrCL band was transected first. We expected that with tibial thrust force applied, we would appreciate measurable cranial tibial displacement; however, we did not detect statistically significant displacement using either CrCL length or ICD. We suspect that our visual inspection and transection of the craniomedial CrCL band may not have been precise enough to completely transect all of the craniomedial band fibers. Even a few intact craniomedial band fibers might be sufficient to prevent tibial subluxation during application of tibial thrust force. Another possibility is that our measurement technique may not be accurate enough to quantify small amounts of cranial tibial displacement present in a partial CrCL transection condition. It is also possible that the tibial thrust force that was applied to the cadaver limbs may not have been sufficient force to induce cranial tibial displacement with the CrCL partially intact.

We chose to evaluate the ICD at three stifle flexion angles: 90°, 110°, and 135°. The 90° stifle flexion angle is the angle that most tibial plateau leveling osteotomies are obtained and represents full stifle flexion. The average stifle flexion angle in standing dogs has been identified as approximately 135° [18]. The 110° stifle flexion angle was chosen as a third angle approximately midway between the flexion (90°) and standing (135°) angles. Based on our analysis, the ICD measurement is an accurate technique for assessing the magnitude of cranial tibial subluxation associated with a status change in the CrCL (Intact to partial transection or partial to complete transection) at any of the three evaluated stifle flexion angles. We recommend performing the ICD measurement at 90° because we found it easiest to induce cranial tibial subluxation at this angle. Regardless of which stifle angle is utilized for tibial subluxation assessment based on ICD calculation, it is important that the same stifle flexion angle be maintained between measurements on the same stifle joint, as the magnitude of the measured ICD changes with changes in stifle flexion angle.

Radiographic techniques for assessment of cranial tibial subluxation in the canine stifle have been previously described [14, 19]. These techniques utilize anatomic landmarks that may be obscured by osteoarthritic changes within the canine stifle. In particular, the caudal margin of the tibial plateau is most affected by the presence of osteophytes [20]. The ICD measurement reported in this study was obtained by measuring the distance between the TMA and FCA. Clinical studies evaluating osteophytosis within the stifle joint noted that osteophytosis does not affect the identification of the intercondylar eminences which are both utilized in the determination of the TMA [20, 21]. Additionally, calculation of the FCA described in our study should not be affected by degenerative changes in the stifle joint. We postulate that the ICD measurement will be reproducible in the presence of osteoarthritis, although additional studies are warranted to test this assumption, since radiographically normal stifles were utilized for this study. One study utilized the center of the femoral condyles, similar to our study, to determine cranial tibial subluxation, but at a stifle angle of 135° only [19]. In contrast, we investigated our ICD measurement at three stifle angles, including flexion and various degrees of extension.

The ICD measurement technique accurately quantified the magnitude of cranial tibial displacement that occurred during a CrCL transection status change. For clinical application, an ICD measurement on the stifle with an intact CrCL would be required prior to ICD measurement in a CrCL rupture condition in order to assess the magnitude of cranial tibial displacement present in a stifle with a CrCL rupture using our technique. If the contralateral stifle has an intact cranial cruciate ligament, the normal ICD measurement could be determined from that limb. Future studies could be aimed at defining normal ICD measurements in various breeds of dogs without cranial cruciate ligament insufficiency, similar to the work that has been done to quantify normal joint reference angles in various breeds of dogs for use in angular limb deformity correction. Reference data obtained from future studies could be used as a standardized normal for ICD values and could allow ICD measurements to be applied more readily to clinical cases with CrCL rupture.

There are several limitations to this study. Our study was an in vitro study using cadavers without normal muscle tension, and may not mimic a live dog in a clinical setting. In our study, the quadriceps muscle group was transected. Other studies evaluating cranial cruciate ligament disease have utilized a spring to simulate the function of the quadriceps mechanism [22, 23]. This was not performed in our study. We used radiographically and orthopedically normal stifles, and it may be more difficult to measure ICD in stifles affected by cranial cruciate disease. Achieving cranial displacement of the tibia was dependent on manual force exerted by the examiner, and the amount of force applied in this study was not standardized, although an attempt was made by the examiner to apply the same thrust force to each stifle. Finally, we only investigated 3 stifle flexion angles in this study and we do not have enough data at this time to know if our findings on the validity of the ICD measurement apply at other stifle flexion angles besides those we evaluated.

## Conclusions

In conclusion, we partially accept our hypothesis as the ICD measurement technique was able to quantify tibial displacement at stifle flexion angles 90°, 110°, and 135° in the intact and completely transected CrCL conditions. We were not able to detect a significant cranial tibial displacement with partial transection of the CrCL with either the CrCL length or ICD measurements. The ICD measurement technique is accurate at all evaluated stifle flexion angles; however, a stifle angle of 90° is recommended due to ease of inducing cranial tibial displacement. Additional studies are indicated to quantify normal ICD measurements in different breeds of dogs for future clinical use.

## Additional file

Additional file 1: **Table S1.** Averaged data of radiographic measurements by observers. (PDF 237 kb)

## Abbreviations

ANOVA: Analysis of variance
CDT: Cranial drawer test
CrCL: Cranial cruciate ligament
FCA: Femoral condylar axis
ICD: Intercondylar distance
PLA: Polylactic acid
SD: Standard deviation
TCT: Tibial compression test
TMA: Tibial mechanical axis

## Footnotes

1 PDS II, Ethicon Endo-Surgery, Somerville, New Jersey, USA.

2 APR-Vet, Sedecal USA, Buffalo Grove, Illinois, USA.

3 Gigabot PLA Filament 3 mm, re:3D, Inc., Austin, Texas, USA.

4 Acukal, J2 Medical, Pittsburgh, Pennsylvania, USA.

5 Orthoplan Elite, Sound-Elkin- a VCA Antech Company, Carlsbad, California, USA.

6 Microsoft Excel, Microsoft Office 2013, Microsoft Corporation, Redwond, WA, USA.

7 Sigmastat, SPSS Inc., Chicago, Illinois, USA.
