# Supplementary material for: Ex vivo preliminary investigation of radiographic quantitative assessment of cranial tibial displacement at varying degrees of canine stifle flexion with or without an intact cranial cruciate ligament
Source: BMC Vet Res. 2018 Sep 3;14:270. doi: 10.1186/s12917-018-1599-5 (PMC6122192; doi:10.1186/s12917-018-1599-5)
Supplement: Supplementary file 1 — Table S1. Averaged data of radiographic measurements by observers. (PDF 237 kb) [file 12917_2018_1599_MOESM1_ESM.pdf]

Table S1. Averaged dataset of radiographic measurements by observers.

Cadaver	CrCL status	Stifle angle	CrCL Length	Length of medial tibial condyle	ICD	CrCL length normalized (tibia)	ICD normalized (tibia)
A	I	90	15.6	19.3	6	0.808311856	0.310540163
A	I	110	15.45	18.75	4.55	0.824324324	0.243598862
A	I	135	16.1	19.4	4.2	0.829839245	0.216128604
A	P	90	15.8	19.35	6.55	0.816516212	0.338323273
A	P	110	16.35	19.25	5.25	0.849595142	0.273211876
A	P	135	16.75	19.25	4.3	0.870169473	0.223513061
A	C	90	18.75	19.15	9.65	0.979139398	0.504022142
A	C	110	21.75	19.25	10.35	1.129924978	0.537645725
A	C	135	23.85	19.2	11.85	1.242234761	0.617136424
B	I	90	15.6	20.15	4.75	0.774161371	0.235739619
B	I	110	15.8	19.9	4.55	0.794201581	0.229095639
B	I	135	16.2	20.15	3.45	0.804347291	0.1716133
B	P	90	18.85	20.3	8.15	0.929854587	0.402167844
B	P	110	18.7	20.2	7	0.926108374	0.34686175
B	P	135	17.3	20.25	4.6	0.854460323	0.227308199
B	C	90	18.65	20.15	8.2	0.925717945	0.407110487
B	C	110	22.35	20.45	11.3	1.09352432	0.552812186
B	C	135	20.35	20.2	8.3	1.007425743	0.410891089
C	I	90	16.65	20.05	4.65	0.830460199	0.231952736
C	I	110	16.95	20.15	4.25	0.841293103	0.210948276
C	I	135	18.15	20.15	3.25	0.900891582	0.161334417
C	P	90	17.8	20.1	5.6	0.885572139	0.278606965
C	P	110	17.65	20.25	4.25	0.871579769	0.209896113
C	P	135	17.35	20.15	3.4	0.861004384	0.168673957
C	C	90	23.25	20.2	12.2	1.150990099	0.603960396
C	C	110	22.6	20.2	11.05	1.118765777	0.54710438
C	C	135	23	20.3	10.85	1.132983887	0.534459328
D	I	90	23.1	24.7	7.1	0.935287175	0.287699974
D	I	110	23.65	24.45	6.7	0.967485863	0.274475524
D	I	135	24.1	24.45	5.4	0.985697558	0.221119103
D	P	110	24.75	24.65	6.3	1.00410632	0.255636932
D	P	135	25.5	24.5	3.55	1.040816327	0.144897959
D	C	90	27.85	24.3	12.35	1.146015751	0.508518926
D	C	110	30.2	24.4	12.5	1.237675317	0.512404468
D	C	135	24.65	24.05	5.5	1.025190246	0.230015566
E	I	90	17.85	23.85	6.85	0.748523207	0.287262658
E	I	110	18.15	23.95	5.9	0.757836471	0.246408647
E	I	135	18.8	23.95	3.65	0.784998257	0.152475593
E	P	90	17.95	24.35	7.45	0.73718165	0.306282678
E	P	110	18.35	24.05	5.05	0.763010478	0.209896608
E	P	135	18.55	24	2.85	0.772916667	0.11875
E	C	90	19.85	24.25	9.55	0.818564432	0.394015917
E	C	110	20.3	23.9	7.1	0.8495019	0.297091934
E	C	135	20.35	24.1	6.65	0.844369835	0.27572314
F	I	90	20.1	27.45	7.45	0.732367541	0.27160756
F	I	110	21.5	27.9	6.25	0.771552001	0.224558674
F	I	135	22.5	27.6	4.8	0.815742115	0.174760597
F	P	90	20.55	27.85	7.95	0.738021559	0.285980453
F	P	110	21.6	27.75	7.75	0.778816928	0.279908035
F	P	135	22.1	27.35	6.25	0.808269822	0.229562776
F	C	90	24.95	27.65	12.35	0.902564103	0.447847985
F	C	110	25.7	27.7	11.45	0.92831158	0.414607591
F	C	135	23.9	27.8	8.5	0.860055139	0.307213399
G	I	90	16.95	26.9	7.5	0.630111524	0.278810409
G	I	110	18.25	27.25	6	0.669757326	0.220197694
G	I	135	19.35	27.05	3.8	0.71532732	0.140440071
G	P	90	18.65	27.25	8.35	0.68447491	0.306502007
G	P	110	19.4	27.4	6.95	0.708011988	0.253619714
G	P	135	20.4	27.1	6.75	0.752818935	0.248638196
G	C	90	20.5	27.2	11.15	0.753663152	0.409610706
G	C	110	24.2	27.6	12.65	0.876811594	0.458333333
G	C	135	23.35	26.9	9.8	0.868062465	0.364524599
H	I	90	18.55	23.35	6.65	0.795096419	0.282286501
H	I	110	19.65	23.8	4.65	0.826648274	0.19422043
H	I	135	20.15	24.7	3.15	0.816882563	0.126180039
H	P	90	18.95	23.25	7.65	0.816732809	0.326960166
H	P	110	20.25	24.35	6.1	0.833189655	0.248935091
H	P	135	20.1	24.5	5.9	0.823095823	0.236691237
H	C	90	19.1	23.75	8.8	0.805039434	0.3665891
H	C	110	20.4	24.05	5.35	0.850097081	0.223380834
H	C	135	23	24.6	8.25	0.935921848	0.331757596
